# Testing and Practical Implementation of a User-Friendly Personalized and Long-Term Electronic Informed Consent Prototype in Clinical Research: Mixed Methods Study

**DOI:** 10.2196/46306

**Published:** 2023-12-19

**Authors:** Evelien De Sutter, David Geerts, Koen Yskout, Stef Verreydt, Pascal Borry, Liese Barbier, Isabelle Huys

**Affiliations:** 1 Clinical Pharmacology and Pharmacotherapy Department of Pharmaceutical and Pharmacological Sciences Catholic University (KU) Leuven Leuven Belgium; 2 Catholic University (KU) Leuven Digital Society Institute Catholic University (KU) Leuven Leuven Belgium; 3 imec-DistriNet Catholic University (KU) Leuven Leuven Belgium; 4 Centre for Biomedical Ethics and Law Department of Public Health and Primary Care Catholic University (KU) Leuven Leuven Belgium

**Keywords:** human-centered design, digital health, qualitative research, informed consent, trial, stakeholders, implementation

## Abstract

**Background:**

Over the years, there has been increasing interest in electronic informed consent (eIC) in clinical research. The user-friendliness of an eIC application and its acceptance by stakeholders plays a central role in achieving successful implementation.

**Objective:**

This study aims to identify insights for the design and implementation of a user-friendly, personalized, and long-term eIC application based on a usability study with (potential) research participants and semistructured interviews with stakeholders on the practical integration of such an application into their daily practice.

**Methods:**

An eIC prototype was evaluated and refined through usability testing among Belgian citizens and iterative redesign. On the basis of a digital literacy questionnaire, a heterogeneous sample of participants was established. Participants needed to complete a series of usability tasks related to personalization and long-term interaction with the research team while using the “think aloud” technique. In addition, usability tests involved completing the System Usability Scale questionnaire and taking part in a semistructured feedback interview. Furthermore, semistructured interviews were conducted with ethics committee members, health care professionals, and pharmaceutical industry representatives active in Belgium and involved in clinical research. Thematic analysis was undertaken using the NVivo software (Lumivero).

**Results:**

In total, 3 iterations of usability tests were conducted with 10 participants each. Each cycle involved some participants who reported having low digital skills. The System Usability Scale scores related to the tasks on personalization and long-term interaction increased after each iteration and reached 69.5 (SD 8.35) and 71.3 (SD 16.1) out of 100, respectively, which represents above-average usability. Semistructured interviews conducted with health care professionals (n=4), ethics committee members (n=8), and pharmaceutical industry representatives (n=5) identified the need for an eIC system that can be easily set up. For example, a library could be established enabling stakeholders to easily provide background information about a clinical study, presented in the second layer of the interface. In contrast, some functionalities, such as informing participants about new studies through an eIC system, were not considered useful by stakeholders.

**Conclusions:**

This study provides insights for the implementation of a user-friendly personalized and long-term eIC application. The study findings showed that usability testing is key to assessing and increasing the user-friendliness of an eIC application. Although this eIC system has the potential to be usable by a wide audience, participants with low digital literacy may not be able to use it successfully, highlighting the need for additional support for participants or other alternatives to an eIC system. In addition, key lessons emerging from the interviews included ensuring that the application is easy to implement in practice and is interoperable with other established systems.

## Introduction

### Background

Digital technologies are rapidly transforming society across many sectors, including the health sector. For many years, the digitalization of health care has been a priority in the European Union [1,2]. One of the objectives of the EU4Health program, adopted as a response to the COVID-19 pandemic, is to strengthen health systems by advancing digital transformation [2,3]. This transformation has the potential to improve patient centeredness and goal-oriented health care [4]. For example, mobile health services may be used to send reminders to patients, monitor patients’ vital signs on a real-time basis, or enable patients to access medical information and communicate with health care professionals [5,6].

The design of digital technologies is one of the critical factors that can influence successful implementation [7,8]. To increase ease of use and adoption, it is crucial to actively involve end users in the design process to translate their knowledge into new ideas and cocreate a technology that meets the end users’ needs [9]. Usability testing is part of the overall user-centered design process, consisting of iterative cycles of understanding the context of use, defining user requirements, and designing and evaluating solutions to meet these requirements. According to the ISO (International Organization for Standardization) standard 9241-11, usability is defined as the “extent to which a system, product or service can be used by specific users to achieve specified goals with effectiveness, efficiency and satisfaction in a specified context of use.” This ISO standard also highlights that the combination of the type of users, goals, and context should be considered in the usability of digital technologies [10]. Usability testing, an established technique in user-centered design, aims to improve user-friendliness by detecting design flaws in interface elements or areas that need further improvement [11].

In the context of clinical research, the informed consent process is considered a basic principle of research ethics [12]. To date, this process has mainly involved paper-based informed consent forms setting forth the pertinent aspects of a clinical study. Owing to the digital transformation, a shift toward electronic informed consent (eIC) has been made. eIC refers to informing research participants and obtaining their consent electronically [13]. It provides research participants with the opportunity to learn about the study through a personalized approach based on their preferences and establish a long-term interaction with the research team during and after the study, for example, to return results or provide participants with new informed consent form versions upon study amendments [14-16]. Throughout this manuscript, the term *personalization* refers to tailoring an eIC application to the research participants’ needs regardless of whether delivering such a personalized user experience is controlled by the application or by the research participants themselves. Digitalizing informed consent may also offer advantages for other stakeholders involved in clinical research. For example, eIC could reduce the administrative burden on research sites or result in fewer inspection findings [17]. Nevertheless, the trend toward a digital future in clinical research requires the reinvention of the modus operandi of these stakeholders [14,18].

### Objectives

To date, various eIC applications, only some of them using personalized and long-term elements, have been developed by for-profit and nonprofit organizations [19-26]. However, only a small fraction of eIC applications have published their usability evaluation results [23-26]. Therefore, this study contributes to the dearth of literature on usability testing of a personalized and long-term eIC, a critical step in its user-centered design process. This study aimed to (1) investigate the usability of a co-designed personalized and long-term eIC prototype with (potential) research participants and (2) seek the views of ethics committee (EC) members, health care professionals, and representatives of the pharmaceutical industry on the practical integration of this eIC prototype into and its influence on their daily practice. The results may support the design of a user-centered eIC application and assist in the responsible adoption of a personalized and long-term eIC application in clinical research.

## Methods

A mixed methods design was used, consisting of (1) usability tests with (potential) research participants and (2) semistructured interviews with EC members, health care professionals, and representatives of the pharmaceutical industry. In both study parts, a prototype implementation of an eIC system was used.

### Prototype Development and Description

**Figure 1:**
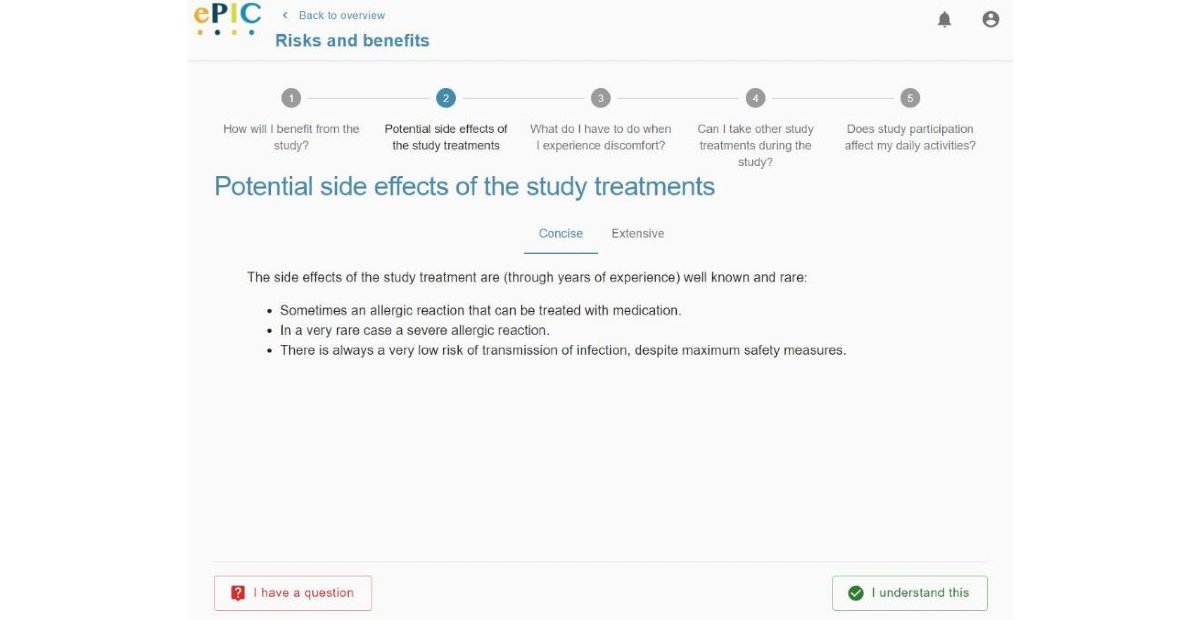
Screenshot of the electronic informed consent prototype in the first iteration—multiple layers of information. Participants can navigate between the concise and extensive layers to hide or access additional information, respectively. If the information is not clear, participants can indicate that they have questions using the buttons at the bottom of the screen.

A user-centered process was undertaken to design an eIC prototype. More concretely, the design of this prototype was informed by a systematic literature review, semistructured interviews with various stakeholder groups involved in clinical research, and a co-design process with participants who had taken part in a clinical study [14-16,27]. On the basis of this research, we identified various elements related to personalization and long-term interaction, such as presenting multiple layers of information or enabling the participants to indicate for which reasons they would like to establish a longitudinal interaction (Multimedia Appendix 1). Only the elements that we believe would add value compared with already existing eIC applications were implemented in the eIC prototype. The prototype, a client-side web application written in React (Meta Open Source), presented information of the Dutch paper-based informed consent form of a clinical study conducted in Belgian hospitals. This prototype has the main feature of providing (potential) research participants with study-related information based on their preferences. To this end, participants can access additional information by consulting the second information layer of the interface (Figure 1). In addition, participants are able to mark study-related information that they have questions about that may be further discussed during a video consultation with a member of the research team. With a particular focus on long-term interaction between the participants and the research team, other features were implemented, such as enabling participants to indicate how and for what reasons they would like to be recontacted (eg, to receive study results), change preferences regarding data sharing, review and sign new informed consent form versions, and withdraw from the study. A detailed description and visualization of the prototype as used in the first iteration of usability testing can be found in Multimedia Appendix 2.

### Usability Testing

#### Participant Selection and Recruitment

Participants for usability testing were recruited via patient organizations and active community outreach, such as local community events. Potential participants were provided with flyers containing information on the eligibility criteria for participants, the course of the study, the compensation for study participation, and the contact information of the researcher. Individuals eligible for inclusion needed to be fluent in Dutch and aged ≥18 years. Those who were interested in participating contacted the researcher to express their interest. Hereafter, interested participants were asked to fill out a survey including demographic questions (eg, age and highest education level) and questions related to digital literacy to establish a heterogeneous sample in the usability evaluation process (Multimedia Appendix 3). This survey, provided to the participants via email or regular mail, was based on the Belgian survey on the use of “Information and Communication Technologies in households and by individuals” [28]. After establishing a heterogeneous sample, the researcher provided the selected participants with the paper-based informed consent form for this study. All participants provided written informed consent before taking part in the usability test. For each iteration, other participants were recruited to ensure that they did not have any experience with the previous eIC prototype.

#### Procedure and Data Collection

Iterative cycles of the usability tests were used to evaluate and refine the eIC prototype following each cycle. After each iteration, the findings were discussed with the research team to further refine the prototype. The usability tests took place between April 2022 and July 2022 and lasted up to 60 minutes. Depending on the preference of the participants, usability tests were conducted face-to-face or using Microsoft Teams (Microsoft Corp). For remote usability testing, the participant was given control of the researcher’s screen to interact with the eIC prototype. At the start of each usability test, the context and aims of this study were explained. In addition, some instructions were provided to the participants (Multimedia Appendix 4). Hereafter, participants were asked to perform specific tasks—without any preacquired knowledge of the eIC prototype—that users may perform when using the application in practice. The given tasks, related to personalization and long-term interaction, varied slightly between iterations depending on the changes made to the prototype (Multimedia Appendix 5). The tasks prepared for the first iteration were tested in 3 pilot usability tests. For a remote usability test, the participant was provided with the tasks via email or regular mail on the days leading up to the usability test. Participants were encouraged to think aloud while performing these tasks, meaning that they had to state out loud and explain their actions. Gaining insights into their reasoning when undertaking specific actions may result in redesign recommendations [29]. During each usability test, we collected performance measures such as the number of errors and moments of confusion. In addition, subjective measures were collected (eg, participants’ spontaneous comments). After each task, we had focused discussions, for example, about specific errors made. After the tasks on personalization as well as the tasks on long-term interaction, the participants filled out the System Usability Scale (SUS). The SUS contains 10 statements for which participants must indicate the strength of their agreement, ranging from 1 (*strongly disagree*) to 5 (*strongly agree*) [30]. Audio and screen recordings were taken during each usability test.

#### Data Analysis

During each iteration, a summary sheet was created containing the number and type of errors made by participants and the number of times they needed help (if applicable). This summary sheet, combined with listening to and watching the audio and screen recordings, respectively, informed the redesign of the eIC prototype. To be able to report the findings adequately, thematic analysis was used for the qualitative data [31]. First, all the audio recordings were transcribed verbatim. Second, the NVivo software (Lumivero) was used to code the first 2 transcripts thematically using a mixed deductive-inductive approach. The creation of deductive codes was based on the tasks, whereas inductive codes focused on the functionalities of the eIC prototype that needed to be redesigned. Hereafter, deductive and inductive codes were categorized, resulting in a working analytical framework. Subsequently, this framework was applied to the other transcripts and was then further refined. Finally, the coded data were charted using Microsoft Excel to create a framework matrix.

The SUS was scored according to the best practices described by Brooke [30], with the score for each statement ranging from 0 to 4. The score for odd statements (ie, statements 1, 3, 5, 7, and 9) is calculated as the scale position minus 1. For even statements (ie, statements 2, 4, 6, 8, and 10), the score is calculated as 5 minus the scale position. The sum of both scores is then multiplied by 2.5 to obtain the overall SUS value between 0 and 100 [32]. Hereafter, the overall SUS value was categorized using adjective ratings ranging from *worst* to *best imaginable* [32,33].

### Semistructured Interviews

#### Interviewee Selection and Recruitment

Semistructured interviews were conducted with people from 3 stakeholder groups: EC members, health care professionals, and pharmaceutical industry representatives. Interviewees were eligible for inclusion if they (1) were involved in clinical research, (2) had fluent proficiency in Dutch, and (3) were active in Belgium. A purposive sample of interviewees was identified by exploring stakeholder websites and via the network of the research group. In addition, snowball sampling was used, whereby interviewees suggested potential recruits. An invitation including the informed consent form for this study was mailed to suitable interviewees. Recruitment continued until data saturation was achieved, meaning that no new insights were observed in the data. All stakeholders provided written informed consent before taking part in an interview.

#### Procedure and Data Collection

Interviews were conducted with multiple stakeholders to explore qualitative insights regarding the practical implementation of the eIC prototype in their daily practice. The interviews took place between June 2022 and September 2022 and were conducted using Microsoft Teams. The interviews were conducted in Dutch, lasted between 23 and 50 minutes, and were digitally audio recorded. All the interviews were conducted by the same researcher (EDS). An interview guide was developed based on the research aims (Multimedia Appendix 6). At the beginning of each interview, the definition of eIC as issued by the European Medicines Agency was provided to interviewees to familiarize them with the term “eIC” [13]. Hereafter, the interviewee was guided through the eIC prototype as used in the third iteration while being asked questions related to personalization and long-term interaction. In addition, the interview guide included questions on the impact of such a prototype on their daily practice.

#### Data Analysis

The analysis of the semistructured interviews was similar to the aforementioned qualitative analysis of the usability tests [31]. However, the creation of deductive codes was informed by themes integrated into the interview guide, whereas inductive codes were created based on observed patterns.

### Ethics Approval

Ethics approval for this study was obtained from the Ethics Committee Research Universitair Ziekenhuis and Katholieke Universiteit Leuven (S66313).

## Results

### Participant Characteristics

In total, 3 iterations of usability testing were conducted with 10 participants each (30 in total). Each iteration involved some participants who reported having little experience with computers or tablets (Table 1). In addition, 15 interviews were conducted with EC members (n=8, of whom n=2, 25% were present at a single interview), health care professionals (n=4), and representatives of the pharmaceutical industry (n=5, of whom n=2, 40% were present at a single interview). All investigators were based in the same hospital. EC members and representatives of the pharmaceutical industry were active in 5 different Belgian ECs and 3 different companies, respectively. We first present the main results on personalization and long-term interaction emerging from the usability tests and the semistructured interviews followed by stakeholder views on other practical elements related to eIC implementation.

**Table 1:** Demographic and computer use characteristics of participants involved in usability testing (N=30).

Characteristic			Iteration 1 (n=10), n (%)		Iteration 2 (n=10), n (%)		Iteration 3 (n=10), n (%)
**Age range (y)**							
	18-39	3 (30)		2 (20)		2 (20)	
	40-59	2 (20)		0 (0)		3 (30)	
	60-79	5 (50)		8 (80)		5 (50)	
**Sex**							
	Male	7 (70)		7 (70)		6 (60)	
	Female	3 (30)		3 (30)		4 (40)	
**Education**							
	Secondary school not completed	0 (0)		0 (0)		1 (10)	
	Secondary school	3 (30)		3 (30)		6 (60)	
	Bachelor’s degree	4 (40)		2 (20)		2 (20)	
	Master’s degree	3 (30)		3 (30)		0 (0)	
	Other	0 (0)		2 (20)		1 (10)	
**Employment status**							
	Student	0 (0)		1 (10)		0 (0)	
	Employed (full time or part time)	5 (50)		1 (10)		5 (50)	
	Retired	5 (50)		8 (80)		5 (50)	
**Having a computer or tablet at home with internet access**							
	Yes	9 (90)		10 (100)		10 (100)	
	No	1 (10)		0 (0)		0 (0)	
**Purposes for which this computer or tablet is used** **(multiple answers possible)**							
	Internet banking	8 (80)		9 (90)		6 (60)	
	Seeking information	9 (90)		9 (90)		8 (80)	
	Sending or receiving emails	8 (80)		8 (80)		7 (70)	
	Checking social media	6 (60)		6 (60)		4 (40)	
	Listening to music	6 (60)		6 (60)		4 (40)	
	Other	3 (30)		3 (30)		2 (20)	
**Frequency of using this computer or tablet**							
	(Almost) every day and even multiple times a day	8 (80)		8 (80)		5 (50)	
	(Almost) every day but not multiple times a day	0 (0)		1 (10)		2 (20)	
	At least once a month but not every week	1 (10)		1 (10)		2 (20)	
	Less than once a month	0 (0)		0 (0)		1 (10)	
**Types of activities carried out in the last 12 months** **(multiple answers possible)**							
	Transferring files between computers, tablets, or other devices	4 (40)		6 (60)		3 (30)	
	Installing software or apps	6 (60)		6 (60)		4 (40)	
	Changing the settings of any programs	2 (20)		0 (0)		1 (10)	
	None of the above	6 (60)		3 (30)		6 (60)	
**Types of software activities carried out in the last 12 months** **(multiple answers possible)**							
	Copying or moving files or folders	6 (60)		7 (70)		4 (40)	
	Using word processing software	7 (70)		7 (70)		5 (50)	
	Creating presentations or documents integrating text, pictures, tables, or charts	5 (50)		4 (40)		1 (10)	
	Using software to edit photos, video, or audio files	4 (40)		3 (30)		2 (20)	
	Writing code in a programming language	1 (10)		2 (20)		0 (0)	
	None of the above	2 (20)		2 (20)		4 (40)	

### SUS Results

The SUS scores for the tasks on personalization (ie, tasks 1-5) and long-term interaction (ie, tasks 6-8) are listed in Tables 2 and 3, respectively. As shown in these tables, the SUS score increased after each iteration. The final average SUS scores were 69.5 (SD 8.35) and 71.3 (SD 16.1) out of 100 for personalization and long-term interaction, respectively. A score of >68 is considered an above-average usability [32].

**Table 2:** System Usability Scale (SUS) scores related to personalization of the electronic informed consent prototype.

Question number	Question	Iteration 1, mean (SD)^a^	Iteration 2, mean (SD)^b^	Iteration 3, mean (SD)^c^
1	I think that I would like to use this system frequently.	3.2 (0.92)	4.2 (0.63)	3.5 (0.71)
2	I found the system unnecessarily complex.	3.2 (0.79)	2.5 (0.85)	2.0 (0.47)
3	I thought the system was easy to use.	3.0 (0.82)	3.5 (1.2)	3.8 (0.63)
4	I think that I would need the support of a technical person to be able to use this system.	2.9 (1.3)	2.8 (1.6)	2.8 (1.2)
5	I found the various functions in this system were well integrated.	3.1 (0.88)	3.7 (0.82)	4.3 (0.48)
6	I thought there was too much inconsistency in this system.	2.0 (0.82)	2.6 (1.4)	2.0 (0)
7	I would imagine that most people would learn to use this system very quickly.	2.7 (0.95)	3.2 (1.1)	3.9 (0.74)
8	I found the system very cumbersome to use.	2.9 (0.74)	2.3 (1.3)	2.1 (0.32)
9	I felt very confident using the system.	3.3 (0.95)	3.4 (1.1)	3.7 (0.68)
10	I needed to learn a lot of things before I could get going with this system.	2.9 (1.1)	2.7 (1.3)	2.7 (0.68)

^a^Total score: mean 53.5 (SD 16.8).

^b^Total score: mean 62.8 (SD 19.4).

^c^Total score: mean 69.5 (SD 8.35).

**Table 3:** System Usability Scale (SUS) scores related to a long-term interaction established through the electronic informed consent prototype.

Question number	Question	Iteration 1, mean (SD)^a^	Iteration 2, mean (SD)^b^	Iteration 3, mean (SD)^c^
1	I think that I would like to use this system frequently.	3.5 (0.85)	4.4 (0.70)	(0.82)
2	I found the system unnecessarily complex.	3.1 (0.88)	2.3 (1.3)	2.1 (0.88)
3	I thought the system was easy to use.	3.3 (1.1)	4.1 (0.88)	3.7 (0.82)
4	I think that I would need the support of a technical person to be able to use this system.	2.7 (1.4)	2.1 (1.3)	2.3 (1.1)
5	I found the various functions in this system were well integrated.	3.6 (1.1)	3.5 (0.85)	4.1 (0.57)
6	I thought there was too much inconsistency in this system.	2.3 (1.2)	2.1 (1.2)	1.7 (0.68)
7	I would imagine that most people would learn to use this system very quickly.	2.7 (1.1)	3.1 (1.2)	3.7 (0.95)
8	I found the system very cumbersome to use.	2.6 (1.1)	2.0 (0.82)	2.0 (0.67)
9	I felt very confident using the system.	3.5 (1.1)	3.6 (0.70)	3.7 (0.68)
10	I needed to learn a lot of things before I could get going with this system.	3.1 (1.2)	2.1 (1.5)	2.6 (1.3)

^a^Total score: mean 57.5 (SD 19.5).

^b^Total score: mean 70.3 (SD 17.3).

^c^Total score: mean 71.3 (SD 16.1).

### Personalization

The personalization features of the eIC prototype concern the display of information at different levels of detail and the handling of questions and unclarities about this information.

#### Usability Testing

##### Overview

The major usability issues related to the personalization features and the changes made after the first and second iterations are summarized in Table 4.

**Table 4:** Major usability issues related to the personalization features and modifications made to overcome them.

Usability issues				Changes implemented	
**Offering information in layers**					
	**Iteration 1**				
		Not being aware that additional information could be retrieved in the second layer of the interface	Add an outlined button including an icon at the bottom of the interface to access this information Add icons to the text buttons “Concise” and “Extensive” at the top		
	**Iteration 2**				
		Not being aware that additional information could be retrieved in the second layer of the interface	Move the topmost buttons “Concise” and “Extensive” to the left of the interface, change to outlined buttons, and change the text label to “Concise information” and “Extensive information”		
	**Iteration 3**				
		Not being aware that additional information could be retrieved in the second layer of the interface	N/A^a^		
**Adding questions and indicating unclear information**					
	**Iteration 1**				
		Not linking the button label “I have a question” to the phrasing “...indicate that you do not understand particular information” mentioned in the task Not indicating the paragraphs containing unclear information	Change the button label to “I do not understand something or have a question” Add the following instruction: “You can select multiple paragraphs. Please click the button ‘Continue’ to continue with the other information sections. By clicking the button ‘Cancel,’ your designations on this page will be removed”		
	**Iteration 2**				
		Not indicating the paragraphs containing unclear information Not knowing how to add a general question^b^	Add clarification in the task Add instructions to the user interface regarding the fact that a general question can be added by pressing the button on the top right of the interface		
	**Iteration 3**				
		Not indicating the paragraphs containing unclear information			N/A
**Providing informed consent**					
	**Iteration 1**				
		Not indicating that all questions were answered	Put the button “Marked as solved” next to the question		
	**Iteration 2**				
		Not correctly using the button “Marked as solved”	Change the button to a slider		
	**Iteration 3**				
		Not correctly using the slider	N/A		

^a^N/A: not applicable.

^b^This task was conducted during the second and third iterations only.

##### Offering Information in Layers

The eIC prototype did not present all the information at once but placed some information in a second layer, requiring an extra interaction to retrieve it. Participants involved in usability testing were asked to consult certain information presented in this second layer. This task revealed usability issues in all iterations. In the first iteration (Figure 1), most participants (6/10, 60%) were not able to find this information promptly when navigating through a particular information section. One participant (P7) said the following:

I did not notice that more detailed information could be consulted.

**Figure 2:**
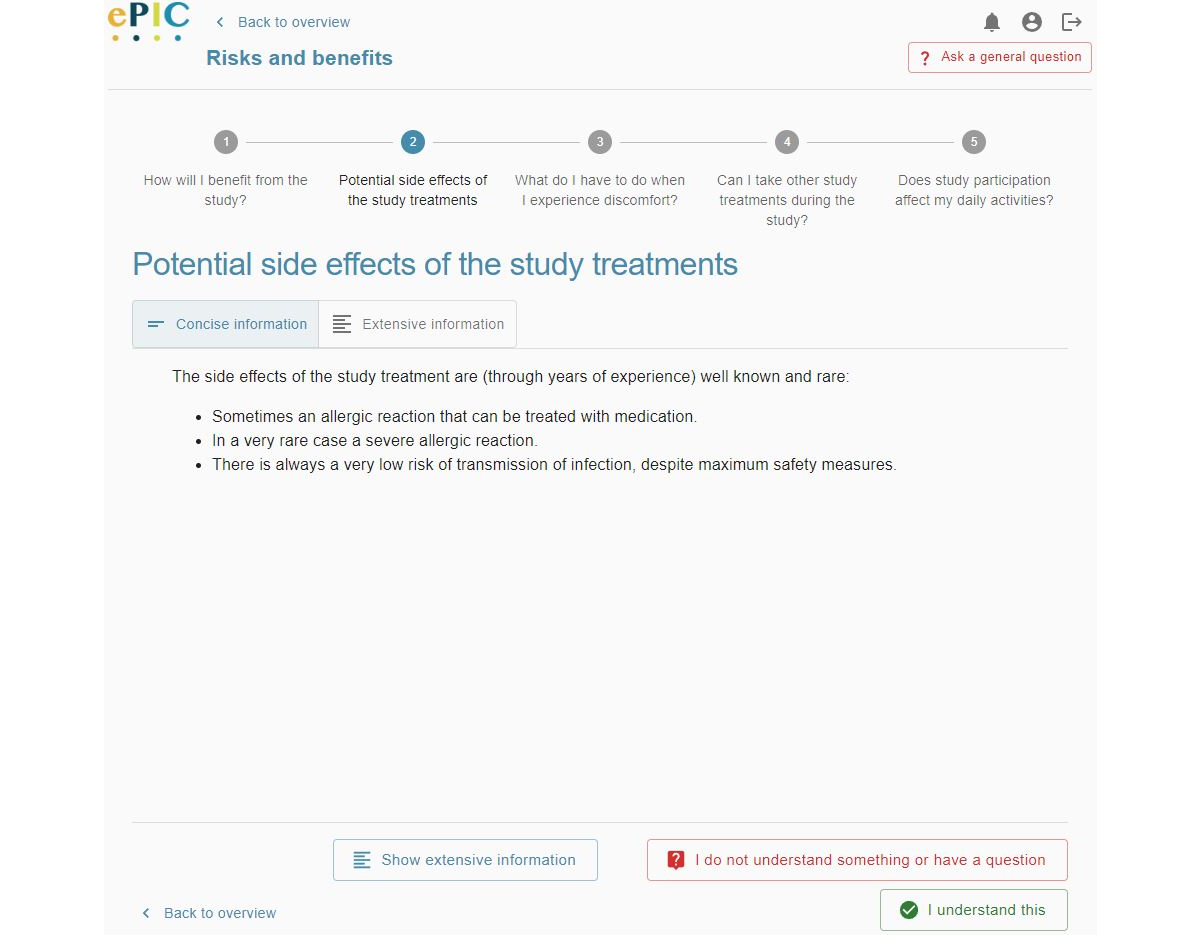
Screenshot of the electronic informed consent prototype in the third iteration—offering information in layers.

Following this iteration, some modifications were made to draw more attention toward this feature, namely by (1) adding an outlined button, including an icon, at the bottom of the interface that participants could also use to switch between concise and extensive information; and (2) adding icons to the text buttons “Concise” and “Extensive” at the top. However, these changes did not have a positive impact on the performance of this task during the second iteration. In this second iteration, multiple participants voiced that “they had not seen that the button ‘Extensive’ was displayed on the interface.” Therefore, it was decided to simulate a tablike interface by moving the topmost buttons to the left side of the interface, using outlined buttons rather than text buttons, and changing the text label of these buttons to “Concise information” and “Extensive information” (Figure 2). As a result of these actions, all but 30% (3/10) of the participants found the required information in the third iteration. In line with the previous iterations, this 30% (3/10) of the participants indicated that they were not aware that additional information could be accessed.

##### Adding Questions and Indicating Unclear Information

The eIC prototype allowed participants to mark information that was not clear to them and add a general question to the investigator in case this question did not correspond to a particular piece of information included in the consent form. Some difficulties with usability were noted for both functions. The main issues observed during the first iteration were that (1) participants did not indicate which paragraph contained unclear information (although the system expected this) and (2) participants did not link the button label “I have a question” to the phrasing “...indicate that you do not understand particular information” mentioned in the task. After completion of the first iteration, the following adjustments were made to the eIC prototype: (1) changing the text label of the button “I have a question” to “I do not understand something or have a question” and (2) adding the following instructions when the button “I do not understand something or have a question” is clicked: “You can select multiple paragraphs. Please click the button ‘Continue’ to continue with the other information sections. By clicking the button ‘Cancel,’ your designations on this page will be removed.”

By making these changes, a positive evolution was noticed in the second iteration. However, there were still some participants who did not indicate the paragraph that contained unclear information. In the second iteration, we also asked participants to add a general question, which was perceived as difficult by most:

I am looking in all information sections but I cannot find how to add this question.

P13

**Figure 3:**
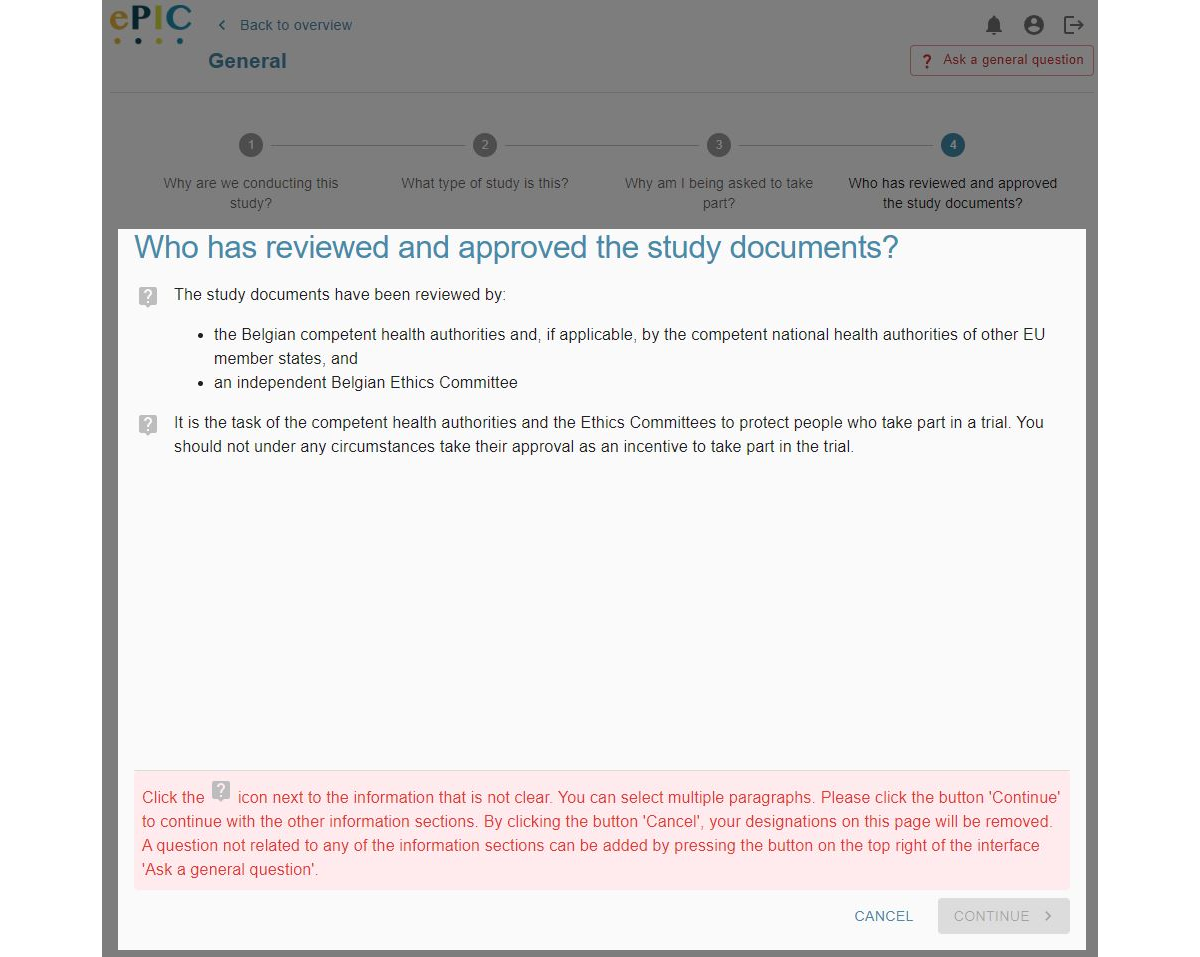
Screenshot of the electronic informed consent prototype in the third iteration—indicating unclear information.

After completion of the second iteration, we clarified in the task that this question was not related to any of the information sections. In addition, the eIC prototype was adjusted as follows: the aforementioned message, which appeared when clicking the button “I do not understand something or have a question,” was highlighted in red and specified that a general question could be added by pressing the button on the top right of the interface (Figure 3). However, in the third iteration, we still observed multiple participants who had difficulty indicating and saving the paragraphs containing unclear information. Specifically, these participants did not know that the question mark icon displayed next to each paragraph needed to be clicked or made use of the button “Cancel” instead of “Continue.”

##### Providing Informed Consent

Participants were asked to provide informed consent. The eIC prototype enforces that all general questions and unclarities be explicitly marked as resolved before consent can be given. Therefore, the participants had to imagine that their general questions and unclear study-related information were discussed with the research team. The major usability problems revealed in the first iteration concerned shortcomings in indicating that all the questions were answered. Following this iteration, the text button “Marked as solved” was placed next to the question or unclarity. Nevertheless, the second iteration revealed that the indication “Marked as solved” was confusing for various participants:

I already clicked “Marked as solved” but it seems that it is not indicated as solved.

P19

**Figure 4:**
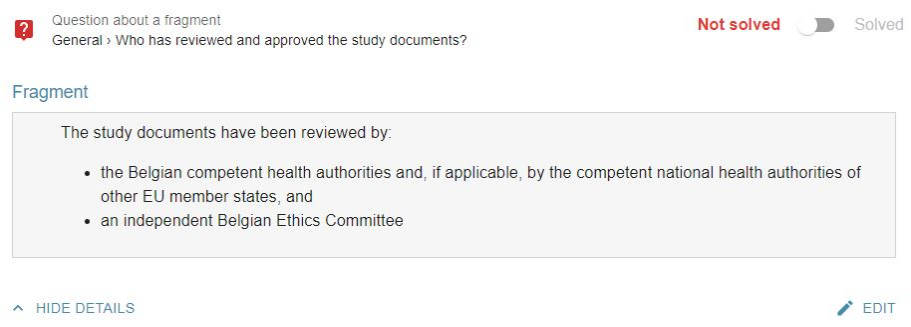
Screenshot of the electronic informed consent prototype in the third iteration—marking questions as resolved.

This error was corrected by implementing a slider, resulting in all but 30% (3/10) of the participants successfully completing this task in the third iteration, mainly as this 30% (3/10) of the participants did not know how to use this slider (Figure 4).

#### Semistructured Interviews

##### Offering Information in Layers

Several interviewees had reservations about this feature. Some pharmaceutical industry representatives mentioned that it could be burdensome for a clinical trial sponsor to provide extensive information. Therefore, they suggested that a library be established containing detailed information on specific health conditions or clinical trial designs. It was also mentioned that an (electronic) informed consent form should, among other things, facilitate the dialogue with the research team and should not be conceived as a document to educate research participants. In addition, pharmaceutical industry representatives were doubtful about the EC members’ and inspectors’ views of the tiered approach:

There is a high chance that inspectors have comments on this functionality, in particular because participants are not obliged to access the extensive information before providing consent.

13; pharmaceutical industry representative

Almost all the EC members were of the opinion that offering information in layers would not be an issue in their assessment process. They highlighted that research participants who would prefer a paper-based informed consent form rather than eIC should be provided with all the information. EC members considered it important to inform the participants, using eIC, of the possibility of accessing additional information if preferred. They also believed that the first information layer should contain all crucial information for participation in the trial, whereas the second layer could further lay down background information. However, an EC member raised the point that it would be challenging to decide which information should be made available in the first and second information layers:

All pertinent aspects of a study should be conveyed to the participants. In case background information is provided, this information can be mentioned under “Extensive information.” However, I think it will be difficult to make a distinction between important information, mentioned in the first layer, and background information.

5; EC member

##### Discussing and Indicating Unclear Information

The interviewees mainly reported that the participants should indicate in the eIC application that their questions have been discussed and answered. However, some health care professionals mentioned that they should also be able to do so in the name of and upon demand of the participant:

If it concerns a person with limited digital literacy, we can go through the eIC system together. In that case, it would be useful that I can also indicate that questions have been answered using my account.

4; health care professional

In addition, it was unclear how and when these questions should be discussed. It was mentioned that this could result in an additional burden for the research team, in particular when questions would be discussed remotely:

If participants read the informed consent form on site, we can immediately discuss any questions. But if the participants would like to discuss questions via a video consultation, does that mean that someone of the research team should always be available?

6; health care professional

##### A Personalized Interface for ECs

A feature that was discussed specifically with the EC members was the possibility of using the eIC platform itself to manage the comments and decisions of the EC rather than an out-of-band mechanism (eg, custom platforms or documents sent via email). Most EC members mentioned several challenges related to this feature. First, it was argued that there are numerous eIC applications that do not offer the same functionalities, negatively affecting a broadly consistent and workable approach. Second, all comments on the trial protocol should be returned in a comprehensive document. To this end, an eIC application should be interoperable with the other systems already in place. Third, it was considered to be burdensome for the patient representatives involved in the EC, who may be older adults, to provide their comments in an eIC application.

##### Additional Functionalities

When interviewees were asked which additional functionalities related to personalization would be needed to add value over paper-based informed consent forms, several suggestions were raised. Health care professionals believed that it would be useful to gain insights into metrics related to a participant’s interaction with the eIC application (eg, the total time interacting with the application). In addition, some pharmaceutical industry representatives mentioned that it would be valuable to know which sections result in the most in questions:

It would be interesting to gain insights into which information sections most often raise questions. I would not say this is a must, but it could help to improve informed consent forms.

14; pharmaceutical industry representative

### Long-Term Interaction

The long-term interaction features of the eIC prototype concern the ability of participants to autonomously change earlier choices (ie, related to sharing their personal data for other research or receiving results of the study), review and sign an updated version of a consent form, and withdraw their consent to participate in the study.

#### Usability Testing

##### Overview

The major usability issues related to the long-term interaction features and the changes made after the first and second iterations are summarized in Table 5.

**Table 5:** Major usability issues related to the personalization features and modifications made to overcome them.

Usability issues				Changes implemented
**Personal data reuse preferences**				
	**Iteration 1**			
		Withdrawing consent for study participation instead of changing preferences on data sharing	Introduce “Withdraw” buttons to undo earlier choices Introduce a confirmation dialogue when informed consent for study participation was requested to be withdrawn	
	**Iteration 2**			
		Withdrawing consent for study participation instead of changing preferences on data sharing	Present the actions of changing preferences on data sharing and withdrawing consent for study participation on 2 distinct interfaces	
	**Iteration 3**			
		Withdrawing consent for study participation instead of changing preferences on data sharing	N/A^a^	
**Changing preferences regarding study results**				
	**Iteration 1**			
		Consulting the “Findings and results” information section instead of clicking the “Manage” button under the “Study preferences” section	Change the title of the section from “Study preferences” to “Study results” Change the text label of the “Manage” button to “Manage your preferences”	
	**Iteration 2**			
		Consulting the “Findings and results” information section instead of clicking the “Manage” button in the “Study preferences” section	N/A	
**New informed consent form version**				
	**Iteration 1**			
		Not being able to recognize the sections containing changes Being confused about how the changes in a new informed consent form version are displayed	Highlight the information sections containing changes in orange Put the text of the previous informed consent form version on the left of the interface while only showing modifications on the right	
	**Iteration 2**			
		Not being able to recognize the sections with changes	Add a “Changed” label in the buttons of the information sections	

^a^N/A: not applicable.

##### Personal Data Reuse Preferences

Participants were asked to indicate that they no longer consented to the use of their personal data in other research and development activities. In the first iteration, changing choices and withdrawing from the study could be done on the same page (Multimedia Appendix 2). Almost half of the participants withdrew their consent for study participation instead of changing their preferences on data sharing. One participant (P5) mentioned the following:

I noticed the button “Withdraw” so I thought that I had to click this button.

**Figure 5:**
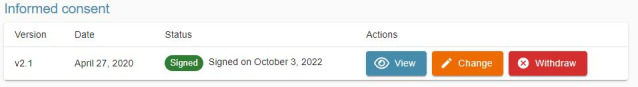
Screenshot of the electronic informed consent prototype in the third iteration—presentation of the actions of changing preferences and withdrawing consent on different interfaces.

Following this iteration, some adjustments were made to the eIC prototype: “Withdraw” buttons were also introduced to undo earlier choices, as well as a confirmation dialogue to verify whether participants wanted to continue or cancel the requested action when informed consent for study participation was asked to be withdrawn. Nevertheless, similar results were observed in the second iteration. Therefore, for the third iteration, it was decided to present the actions of changing preferences on data sharing and withdrawing consent for study participation on 2 distinct interfaces (Figure 5), which positively affected the results. However, there was still one participant who withdrew his consent for study participation.

##### Changing Preferences Regarding Study Results

The participants were requested to change their preferences regarding receiving the study results via email. This could be done in the prototype by clicking the “Manage” button under the “Study preferences” section. The main issue observed in the first iteration related to some participants who instead consulted the “Findings and results” information section of the consent form to change their preferences:

In this section it is mentioned that the results will be made available on specific websites. I believe I have to click these hyperlinks to indicate that I wish to receive the results.

P8

To support end users in changing their preferences regarding study results, we changed the title “Study preferences” to “Study results” and the text label of the button “Manage” to “Manage your preferences.” As a result, all but 10% (1/10) of the participants in the second iteration and all participants in the third iteration were able to successfully change their preferences.

##### New Informed Consent Form Version

**Figure 6:**
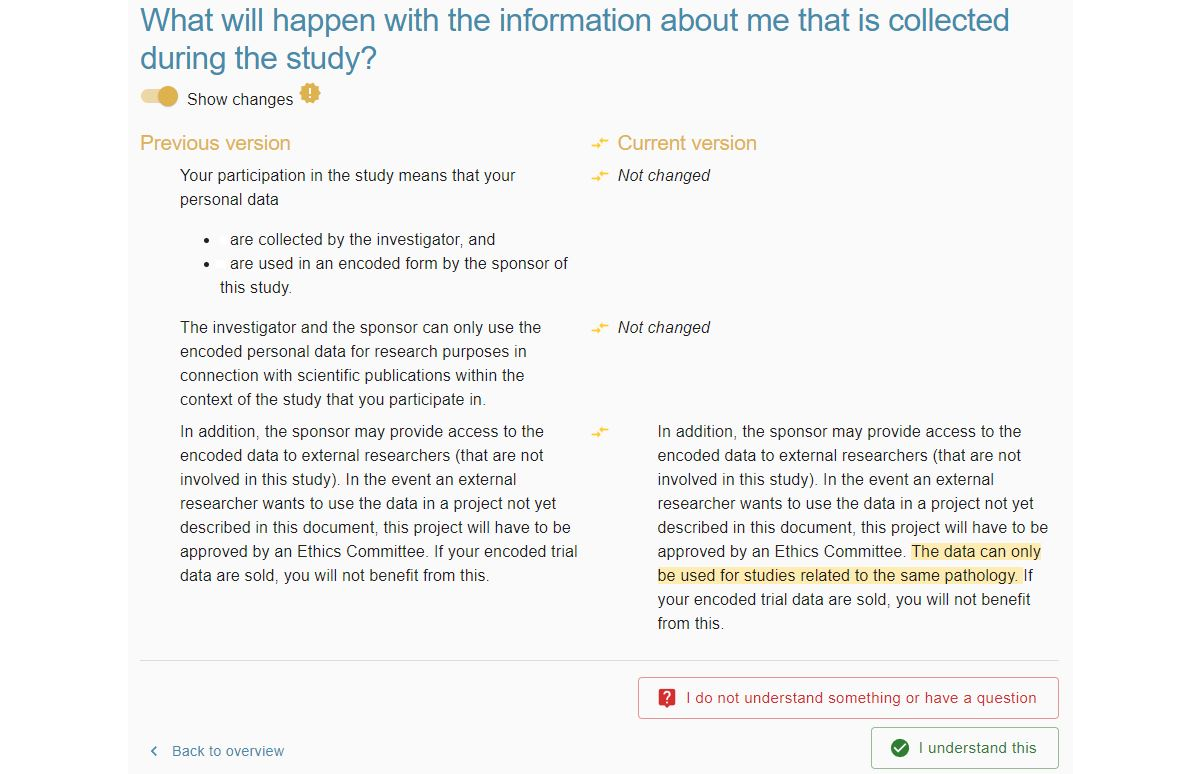
Screenshot of the electronic informed consent prototype in the third iteration—presentation of the concrete changes of a new informed consent form version.

**Figure 7:**
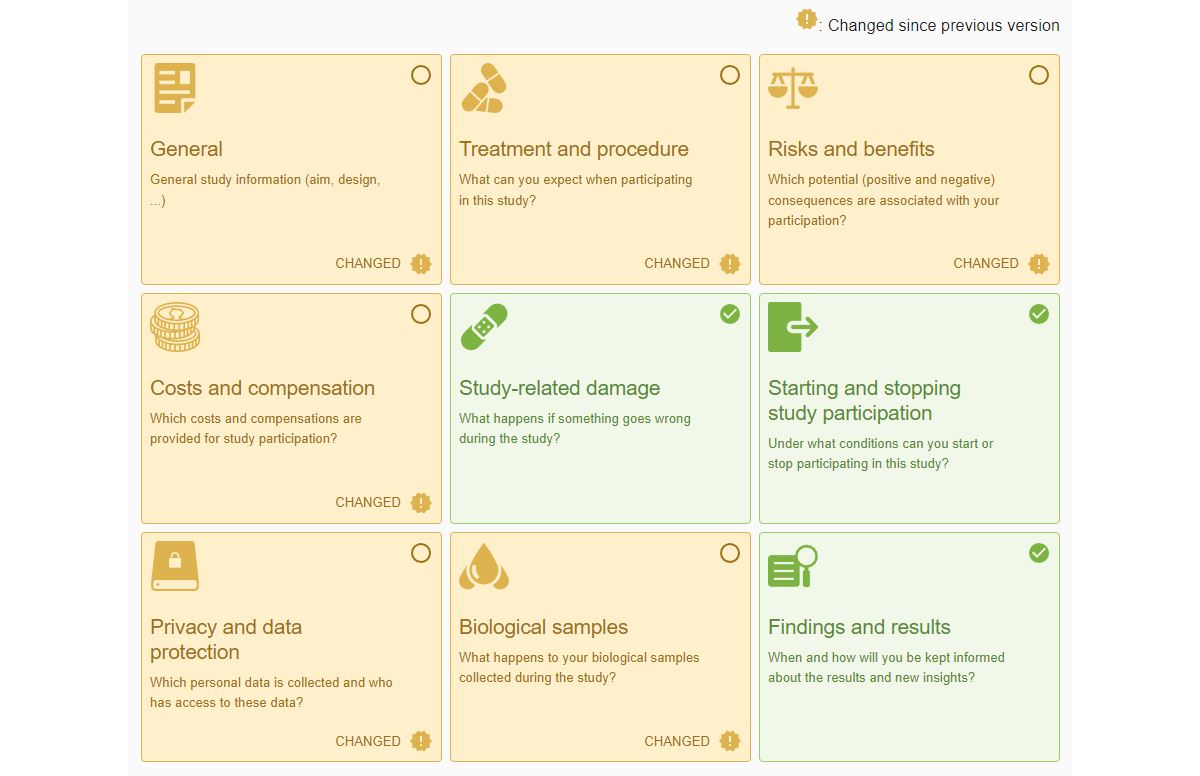
Screenshot of the electronic informed consent prototype in the third iteration—presentation of the information sections of a new informed consent form version.

Participants received a notification that a new informed consent form version had been made available. When viewing the new informed consent form, the eIC prototype indicates which sections have changed and provides a side-by-side view to compare the changes to the text. In the first iteration, unchanged information sections (ie, those identical to the previously signed consent form) were highlighted in green, whereas changed sections had a white background with an icon that indicated that they were changed (Multimedia Appendix 2). Some participants thought that the unchanged information sections highlighted in green were those that included changes. In addition, almost half of the participants were confused about how the concrete changes to the text were displayed. It was mentioned that “the logic was hard to find” (P1) and that “it would be better to put the new version on the right side of the interface” (P3). In response, for the second iteration, we highlighted the information sections containing changes in orange and put the integral text of the previous informed consent form version on the left of the interface while only showing modifications on the right (Figure 6). Nevertheless, one participant (P22) involved in the second iteration mentioned that “it was unclear which sections included changes.” To this end, efforts were made to further emphasize the changed information sections by explicitly adding a “Changed” label (Figure 7), which resulted in all participants successfully completing this task in the third iteration.

#### Semistructured Interviews

##### Withdrawing Trial Participation and Changing Preferences

Some EC members and pharmaceutical industry representatives argued that withdrawing consent should be as easy as giving it and, therefore, agreed that participants should be able to withdraw their consent electronically. In addition, a few pharmaceutical industry representatives voiced that this functionality is in line with the Good Clinical Practice standard and would ensure consent process efficiency by providing an audit trail, which is a reliable record of all consent-related activities. Many interviewees across stakeholder groups mentioned that it is of utmost importance that the participants’ further medical care is discussed with the research team after withdrawing. To this end, it was suggested to organize a conversation with the research team once the button to withdraw is clicked to give the participants a variety of instructions for ending their participation in the trial. For instance, additional visits may be required to monitor the participant for any future adverse events. Some interviewees were reluctant to enable participants to withdraw their consent electronically, in particular as participants may have unwarranted concerns about the study. Therefore, it was suggested that the eIC interface should enable participants to provide their reasons for withdrawing prematurely from the trial and that clicking the button “Withdrawing consent” should not immediately result in permanent withdrawal from the study:

It should be avoided that participants have unwarranted concerns and are therefore withdrawing from the study. I would also not activate the withdrawal immediately but first organize a consultation with the research team to discuss any issues.

2; health care professional

In addition, several health care professionals stated that they should receive an alert when a participant would like to withdraw from a trial, preferably via the participant’s electronic health record:

I am already overloaded with emails. In case of paper-based informed consent forms, the participant informs the investigator or the study nurse orally about withdrawing from the trial. If the participant withdraws his or her consent electronically, I would prefer to be informed via the participant’s electronic health record rather than via email.

4; health care professional

Similarly, questions arose regarding how the research team would be informed of changed preferences (eg, about data sharing) and whether these preferences would be linked to a particular database. Some interviewees also stated that it was key to inform participants about this feature and the potential implications:

There are studies in which the participants should explicitly consent to additional investigations on their samples. These investigations are key to advance scientific research. Therefore, I think it is important to inform participants about the implications of changing their preferences.

16; pharmaceutical industry representative

##### Receiving Information About New Studies

An additional functionality discussed with the interviewees was a feature to inform participants of new studies they could consider participating in. This was not deemed valuable by the interviewees. Some pharmaceutical industry representatives mentioned that this feature is not useful as it is already part of established eSource systems. In addition, some interviewees across stakeholder groups stated that this feature does not belong in an eIC application:

Receiving information about new studies via an eIC system goes too far. I am not in favor of it.

10; EC member

Moreover, questions arose regarding the type of studies that participants would be informed about and whether this functionality would result in revealing the identity of participants who have taken part in a particular trial.

##### Providing Results

Several interviewees raised the point that an eIC application could be used to inform the research participants about the final study results. However, it was mentioned that preliminary results are often not shared for various reasons, such as to avoid creating false expectations or the potential to have increased dropout rates in the control and experimental arms. Therefore, it was not considered useful to have the option of sharing preliminary results in an eIC application. Some interviewees indicated that the return of a clinical trial summary in lay language is a mandatory requirement of the Clinical Trials Regulation and, therefore, questioned the need to explicitly ask participants whether they would like to receive this summary via an eIC application:

Offering the participants the choice will result in logistic issues. Either we do it by default or we inform the participants that they can access the results on the clinical Trials Information System website.

15; pharmaceutical industry representative

In addition, a pharmaceutical industry representative suggested to clearly specify until when participants could change their preferences regarding study results to enable this functionality from an organizational perspective.

### Setup and Interoperability of a Personalized and Long-Term eIC Application

Some interviewees believed that interoperability, for example, with electronic health records or eSource systems, is a key enabler of successful implementation of eIC. An EC member also voiced the opinion that interoperability between the eIC application and the Clinical Trials Information System would be valuable:

It would be useful if the eIC system could communicate with the Clinical Trials Information System. However, I doubt the feasibility because it concerns a European system.

5; EC member

According to some pharmaceutical industry representatives, it would be challenging to develop a personalized and long-term eIC application. Therefore, they suggested that a template, which already offers the building blocks, be developed. Similarly, a health care professional mainly involved in phase-1 trials mentioned that there are often changes in the informed consent form because of study amendments. To this end, the ability to quickly set up and modify the eIC application was deemed paramount.

## Discussion

### Principal Findings

This study aimed to identify insights for the design and implementation of a user-friendly personalized and long-term eIC application in clinical research based on a usability study with (potential) research participants and semistructured interviews with various stakeholder groups (ie, EC members, health care professionals, and pharmaceutical industry representatives). The eIC prototype, evaluated in the third iteration of usability testing, scored 69.5 (SD 8.35) and 71.3 (SD 16.1) out of 100 on the SUS for personalization and long-term interaction, respectively, rating the overall usability as above average [32]. Overall, stakeholders reported divergent views with regard to the eIC prototype, for example, related to the functionality of withdrawing consent to take part in a study through an eIC system. Furthermore, they emphasized the importance of an eIC system that can be easily set up and implemented in practice.

### Improving the Participants’ Understanding

In designing the eIC prototype, we aimed to include (1) a tailored approach based on the individual’s information needs and (2) the potential to establish a longitudinal interaction with the research team. Our eIC prototype included similar information to that included in a paper-based informed consent form of a trial conducted in Belgian research sites. However, this information was presented in another format, for example, using expandable sections and a layered approach, allowing for the distillation of information to meet individual participant needs. In this study, emphasis was mainly placed on functionalities related to personalization and long-term interaction rather than on enhancing the participants’ understanding. A systematic literature review conducted by Pietrzykowski and Smilowska [34] indicated that study participants’ deficiencies in understanding are primarily related to not grasping the concept of randomization and placebo as well as the risks associated with study participation. By using interactivity and multimedia, eIC may contribute to a better understanding of study-related information [35,36]. To this end, efforts should be made to present specialized medical and legal terminology in a way that meets the unique learning needs of patient populations and, thus, facilitate informed decision-making.

### Leveraging a Personalized and Flexible eIC System

A few participants in the third iteration of usability testing had difficulty completing the tasks related to the personalization features of the eIC prototype, for example, to access information presented in the second layer of the interface, potentially compromising a valid consenting process. However, EC members highlighted that all the crucial information should be part of the first information layer, thus overcoming the ethical issue of research participants not being fully informed about what it means for them to take part in a clinical study. Furthermore, if research participants would like to learn more about a particular topic, a consultation with the research team could be set up.

In addition to usability testing, the likelihood that an eIC system will be accepted by stakeholders in the field is a prerequisite for successful adoption [37]. Several comments from stakeholders indicated a hesitant attitude toward our proposed eIC format. For example, during the semistructured interviews, it was mentioned that an informed consent form should not be conceived as a document to educate research participants, thereby eliminating the need for providing background information in the second layer of the interface. Although there is no one definition of informed consent, it usually includes that participants agree to take part in a study “based on an understanding of (usually disclosed) relevant information,” which can only be possible if they are sufficiently educated [38]. Other issues observed during usability testing relate to indicating paragraphs that contain unclear information and marking questions and unclarities as resolved, which is a mandatory step in the eIC prototype before being able to consent. However, for the latter, health care professionals mentioned that they should also be able to indicate that these questions have been answered on behalf of the participant, thus mitigating the impact of this usability issue.

To minimize the aforementioned usability issues in practice, we propose the design and use of a personalized and long-term eIC system to be centered on the following recommendations: (1) pay careful attention to making the existence of additional information readily apparent on the eIC interface, (2) inform research participants about the opportunity to access additional study-related information by consulting the second information layer of the interface, (3) prefer interfaces that lead participants to record superfluous questions rather than accidentally removing questions, and (4) inform research participants that (general) questions can be added and clarify how to do so and how these can be resolved after discussion with the research team.

In addition to personalization features for research participants, an eIC system could also provide ECs with a personalized interface to manage their comments and decisions. However, one of the challenges mentioned by EC members regarding this feature concerns interoperability with already established systems. An eIC application being isolated from the existing infrastructure, resulting in an additional workload for stakeholders, should be avoided [39]. To this end, it is key that an eIC application can be integrated into existing IT systems to enable seamless communication across these systems [40]. In the context of the ethics review, software could be leveraged to automatically generate comprehensive documents (potentially in multiple formats) including all comments on the informed consent form and the trial protocol provided electronically by the EC members, thereby increasing interoperability without increasing EC members’ workload.

### Design Considerations for Driving Long-Term Interaction Features

The developed eIC prototype includes various long-term interaction features: research participants are able to change their preferences with regard to sharing personal data and receiving study results, reconsent upon study amendments, and withdraw their consent to take part in the clinical study. In the third iteration of usability testing, we identified participants who accidentally withdrew from the study, which can seriously hinder research [41]. Although one might question whether the introduction of additional hurdles is ethically and legally acceptable, stakeholders’ proposal to organize a consultation with the research team when a participant wishes to withdraw from the study may act as a safeguard in this regard. Generally, to minimize usability issues with longitudinal features in real-life clinical situations, the following recommendations can be derived from our study: (1) provide alternatives that prevent participants from accidentally withdrawing their consent (eg, at the beginning of the interface to withdraw consent, first refer to the possibility of changing preferences [such as regarding data sharing] or use a decision aid that ensures that the correct choice is made), (2) inform research participants that they can change preferences with regard to receiving study results, and (3) ensure that the changes in a new informed consent form version are clearly visible. However, even when considering the aforementioned recommendations for personalization and long-term interaction features, additional considerations may be necessary to ensure the inclusion of participants who are unaccustomed to digital interactions in their personal lives. For example, support from the research team may be needed to guide participants through the eIC interfaces, or participants can be given the choice between eIC and a paper-based informed consent form.

### Strengths and Limitations

Participants with varying digital skills were involved in all iterations of usability testing, which can be considered a major strength. However, the usability results should be viewed in the context of the study’s limitations. The eIC prototype was evaluated in a simulation-based setting, and its usability in real-life clinical situations, in which patients may be overwhelmed, may be different. To this end, in situ testing, meaning that end users are observed in live contexts when interacting with the application, may result in additional usability-related barriers [42]. In addition, some usability issues may be because participants did not completely review the study-related information and, therefore, did not fully grasp the process of providing informed consent (ie, reviewing information, discussing questions with the research team, and providing consent). With regard to the semistructured interviews conducted with stakeholders involved in clinical research, it should be noted that the participating stakeholders were mainly active in Belgium. To this end, firm generalizations should not be made from this qualitative study.

### Conclusions

This study aimed to identify barriers to and enablers of the use of a personalized and long-term eIC prototype as well as gather multi-stakeholder insights regarding the practical integration of such a prototype into their daily practice. During iterative usability testing, participants were presented with several tasks that trial participants would typically perform using an eIC application. In summary, our study shows that usability testing is a critical step in the development of eIC to learn about the gaps in functionality. In addition, a key step toward achieving successful eIC implementation is to understand how stakeholders’ daily practice would be influenced. Overall, the stakeholders involved in the semistructured interviews emphasized the importance of establishing an accessible library and template to enable an efficient setup of a personalized and long-term eIC prototype. In addition, divergent views were identified with regard to withdrawing consent for study participation electronically. The various findings that emerged from this study are useful for the design and implementation of a personalized and long-term eIC system in clinical research.

## Appendix

Multimedia Appendix 1 Elements related to personalization and long-term interaction identified in previous research.

Multimedia Appendix 2 Description of the initial electronic informed consent prototype as used in the first iteration.

Multimedia Appendix 3 Survey including demographic questions and questions related to digital literacy.

Multimedia Appendix 4 Instructions for participants taking part in a usability test.

Multimedia Appendix 5 Tasks for participants taking part in a usability test.

Multimedia Appendix 6 Interview guide.

## Abbreviations

EC: ethics committee
eIC: electronic informed consent
ISO: International Organization for Standardization
SUS: System Usability Scale
