# Supplementary material for: Testing and Practical Implementation of a User-Friendly Personalized and Long-Term Electronic Informed Consent Prototype in Clinical Research: Mixed Methods Study
Source: J Med Internet Res. 2023 Dec 19;25:e46306. doi: 10.2196/46306 (PMC10762617; doi:10.2196/46306)
Supplement: Multimedia Appendix 1 [file jmir_v25i1e46306_app1.docx]

**Multimedia Appendix 1. Elements related to personalization and long-term interaction, identified in previous research**

| **Elements related to personalization and the long-term interaction** |
| --- |
| **1. Presentation of information** *(personalization)****:***  Present a first layer of information that is essential for participation in the research study, followed by a second layer that offers more specific information to those who are interested (for example, by using hyperlinks or by hovering over a word). For example, the definitions of the terms ‘randomization’ or ‘double-blinded’ could be mentioned in the second layer. |
| **2. Providing personalized information** *(personalization)****:***   - Offer the ability for physicians to stratify groups and provide adapted information (e.g., only give information about pregnancies and breastfeeding to women with a childbearing age). - Implement a decision tree for information related to pregnancy, contraception, and interaction with other drugs. By interacting with this decision tree, research participants are able to receive personalized information. |
| **3. Lay-out** *(personalization)***:**  Enable the research participants to change the layout (e.g., font size) and highlight information. |
| **4. Accessing information** *(personalization)***:**  Provide research participants with the option to choose between audio or video content (particularly in case of hearing or visual impairment). |
| **5. Test** *(personalization)***:**  When implementing a test to assess research participants’ comprehension, automatically redirect them to the relevant information/specific topic when they have answered a question incorrectly. Moreover, provide them with the possibility to use the ‘tell me more’ button, before answering the question. |
| **6. Metrics** *(personalization)*  Provide the possibility to implement metrics that allow physicians to monitor various variables (e.g., how long did it take for research participants to read a certain topic). |
| **7. Long-term contact** *(personalization & long-term interaction)****:***  Enable the research participants to indicate if, how, for which duration, and for which reasons they would like to be recontacted (e.g., to receive final study results, results on additional investigations on the participants’ samples, or to receive invitations for new research studies for which participants are being sought). |
| **8. Notifications** *(personalization & long-term interaction)****:***  If research participants would like to be recontacted, enable them to set preferences about the type (e.g., e-mail, text messages) and frequency of notifications. |
| **9. Data sharing** *(personalization & long-term interaction)****:***  Provide research participants with the option to tailor their preferences about whom they want to share their personal data with. |
| **10. Contacting the research team** *(personalization & long-term interaction)****:***  Research participants must have the opportunity to contact the research team if they have questions. Nevertheless, their preferences on how to contact the research team (e.g., via a chatbot, chat feature, or video-consultation) may depend on the importance of their questions (e.g., non-urgent questions may be asked via the chat) and their familiarity with technology. |
| **11. Presenting new versions of electronic informed consent** *(personalization & long-term interaction)****:***  When presenting a new consent version, the changes can be indicated and additional information on the modifications can be presented. In this way, participants have the option on whether or not to access the additional information. |
| **12. Overview of studies** *(personalization & long-term interaction)****:***  Research participants could have the possibility to navigate throughout the electronic informed consent interface to have an overview of studies in which they have previously taken part, accompanied by their results. |
