# Supplementary material for: Testing and Practical Implementation of a User-Friendly Personalized and Long-Term Electronic Informed Consent Prototype in Clinical Research: Mixed Methods Study
Source: J Med Internet Res. 2023 Dec 19;25:e46306. doi: 10.2196/46306 (PMC10762617; doi:10.2196/46306)

**Multimedia Appendix 2. Description of the initial electronic informed consent prototype, as used in the first iteration**

**Interface 1: Sign in to start**

- Multiple options are provided to sign in (e.g., username and password).

**Interface 2: Homepage**

- This interface includes the headings ‘My studies’ and ‘My preferences’.
- Under the heading ‘My studies’, an overview of clinical trials is displayed in which the participant had taken part (marked as ‘Finished’), would like to participate (marked as ‘Action required’), or is already participating. For each trial, the study details can be consulted by clicking the button ‘Open’ (in this case, the participant navigates to interface 3).
- Under the heading ‘My preferences’, a hyperlink is included to navigate to the participant’s profile page (i.e., interface 8).
- The top of this interface contains a notification icon and a profile page icon. When clicking the latter, the participant has the choice to navigate to his/her profile page (i.e., interface 8) or to log out. These icons are also present on the following interfaces.

**Interface 3: Study details of a specific clinical trial**

- This interface includes the headings ‘Contact information’, ‘Informed consent’, ‘My appointments’, and ‘Study preferences’.
- Under the heading ‘Informed consent’, the following is mentioned: version number, date, status (can be marked as ‘To be signed’, ‘Signed’ (accompanied by the date of signing), or ‘Outdated’), and actions (can include the buttons ‘View’ and ‘Change or withdraw’ if the informed consent form has been signed or the button ‘View and sign’ if the informed consent form has yet to be signed). When clicking the buttons ‘View’ or ‘View and sign’, the participant navigates to interface 4. In case of clicking the button ‘Change or withdraw’, the participant is redirected to interface 10.
- The heading ‘Study preferences’ includes the button ‘Manage’ (when clicking this button, the participant navigates to interface 9).

**Interface 4: Overview: signing the informed consent**

- This interface includes the headings ‘1. View information’, ‘2. Answering questions’, and ‘3. Signing’.
- Under the heading ‘1. View information’, clinical trial-related information is displayed in large information sections (i.e., ‘General’, ‘Treatment and procedure’, ‘Risks and benefits’, ‘Costs and compensation’, ‘Study-related damage’, ‘Starting and stopping study participation’, ‘Privacy and data protection’, ‘Biological samples’, and ‘Findings and results’). This information can be accessed by clicking on a particular section or by clicking the button ‘Start’.
- The section in which the participant marked unclarities/questions is highlighted in red. If the participant indicates that all information in a particular section is understood, this section is highlighted in green. If it concerns a new informed consent version, the section that includes changes contains a yellow exclamation mark. Above the sections, the meaning of this exclamation mark is explained. The sections that do not contain changes are highlighted in green.


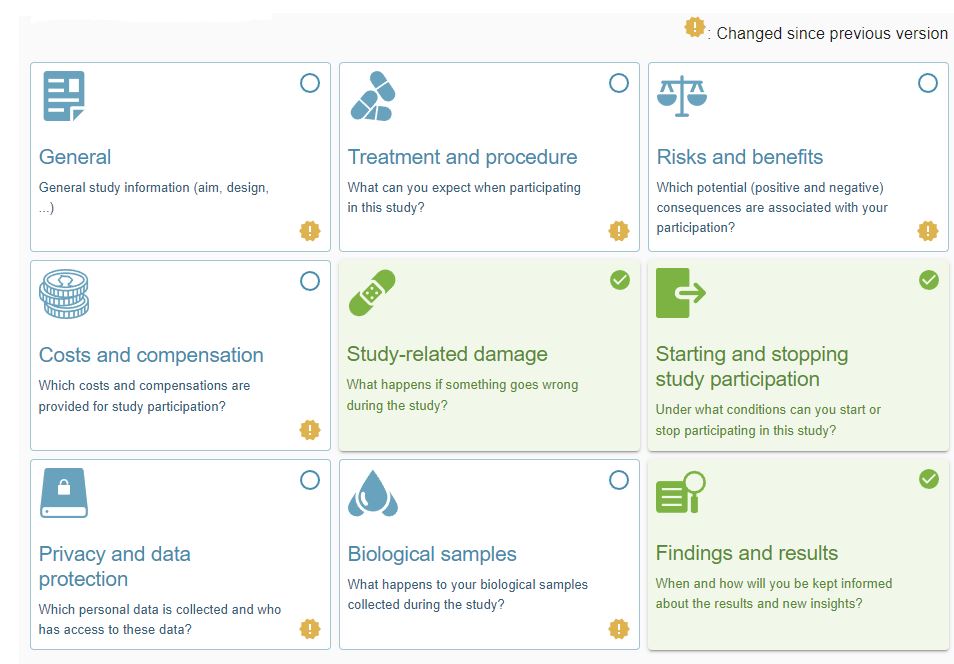


- Under the heading ‘2. Answering questions’, the number of questions added by the participant is mentioned. In addition, the participant is informed that ‘*these questions can be solved via a conversation with one of the members of the study team*’. An overview of the questions can be displayed by clicking the button ‘Open questions’ (in this case, the participant navigates to interface 6).
- Under the heading ‘3. Signing’, the participant is informed that the informed consent can be signed only if the participant has read all the study-related information and if all questions, if applicable, have been answered. The button ‘Sign consent’ is only clickable if these conditions are met (in case this button is clicked, the participant navigates to interface 7).

**Interface 5: A particular information section**

- The information included in a particular section is divided into multiple parts. These parts, visible at the top of the interface, are indicated with numbers and titles. When the participant clicks a particular information section, the first part is displayed. However, the participant has the possibility to view another part by clicking on the abovementioned numbers and titles.
- If it concerns a new informed consent version, the parts are preceded by an overview of the changes. In the parts containing changes, the updated study-related information is displayed on the left side of the interface in which the changes are highlighted in green. The right side of the interface contains the label ‘No changes’ or contains the information of the previous consent version for paragraphs in which changes have been made.


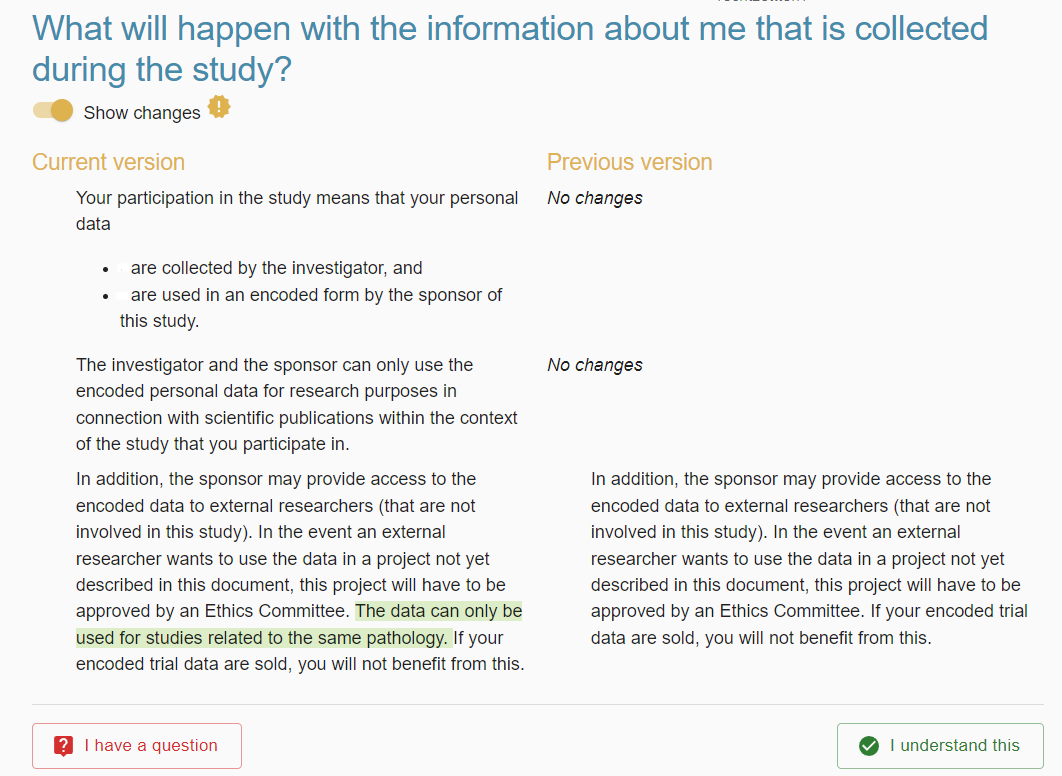


- The bottom of the interface contains the buttons ‘I have a question’ and ‘I understand this information’ on the left and right side of the interface, respectively. If the participant clicks the button ‘I have a question’, the following sentence is displayed ‘*Please indicate above which information is not clear using the question mark icon*’. By using this question mark icon, displayed next to each paragraph, the participant can indicate the paragraph containing unclear information. Hereafter, a pop-up screen appears in which the participant can take additional notes, if preferred. More specifically, the pop-up screen contains the following information ‘*We note that this information is not clear for you. You can discuss this with the investigator later on. If you wish, you can make notes. These notes are also visible for the investigator*’. The participant can use the buttons ‘Remove question’, ‘Cancel’, or ‘Confirm’. If the button ‘I understand this information’ is clicked, the participant is redirected to the following part or the overview (i.e., interface 4) in case it concerns the last part.


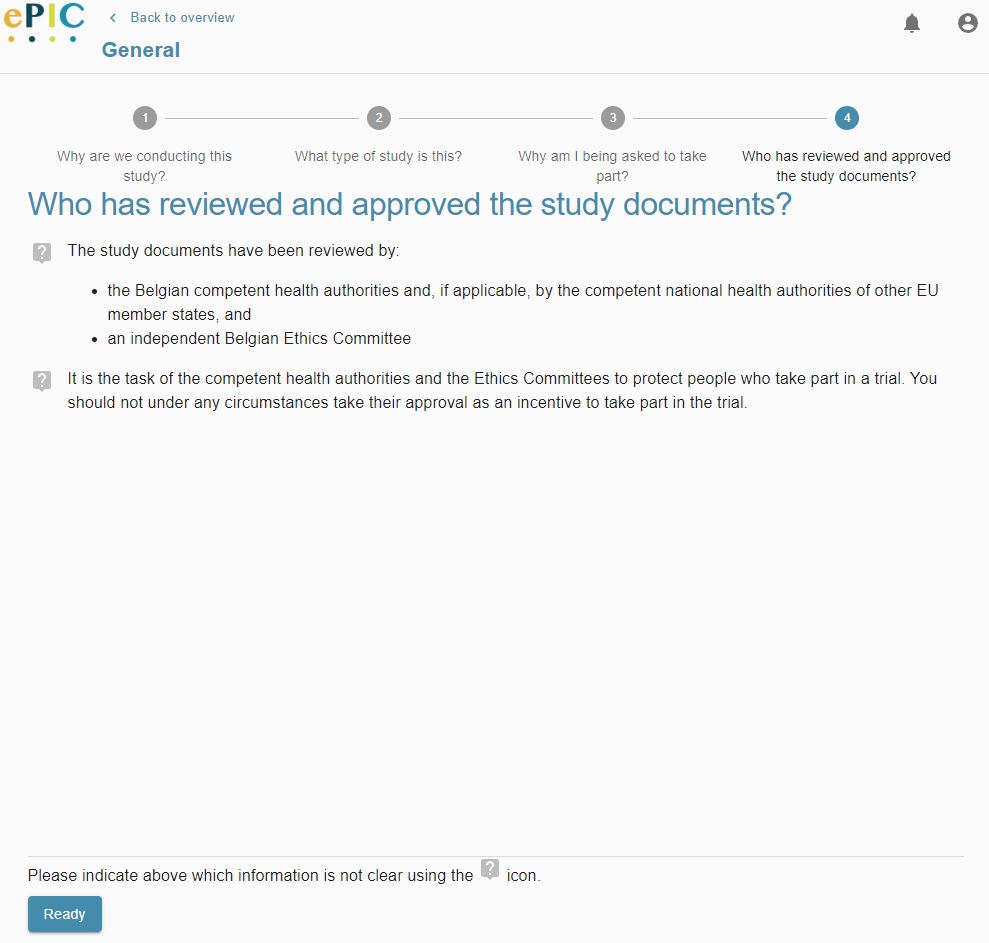


- The ‘i’ symbol is added to some terms, marked in blue, such as adaptive and randomized. When hovering over these terms, a tooltip is shown (e.g., ‘What is a randomized study?’). When clicking a particular term, a popover, containing more details, is displayed. In addition, some study-related information is offered in layers: a first layer offering concise information, followed by a second layer that includes more detailed information. At the top, in the middle of the interface, the text buttons ‘Concise’ and ‘Extensive’ are displayed, which allow the participants to navigate between the two layers. Further information is also offered in dropdown-boxes in particular sections.


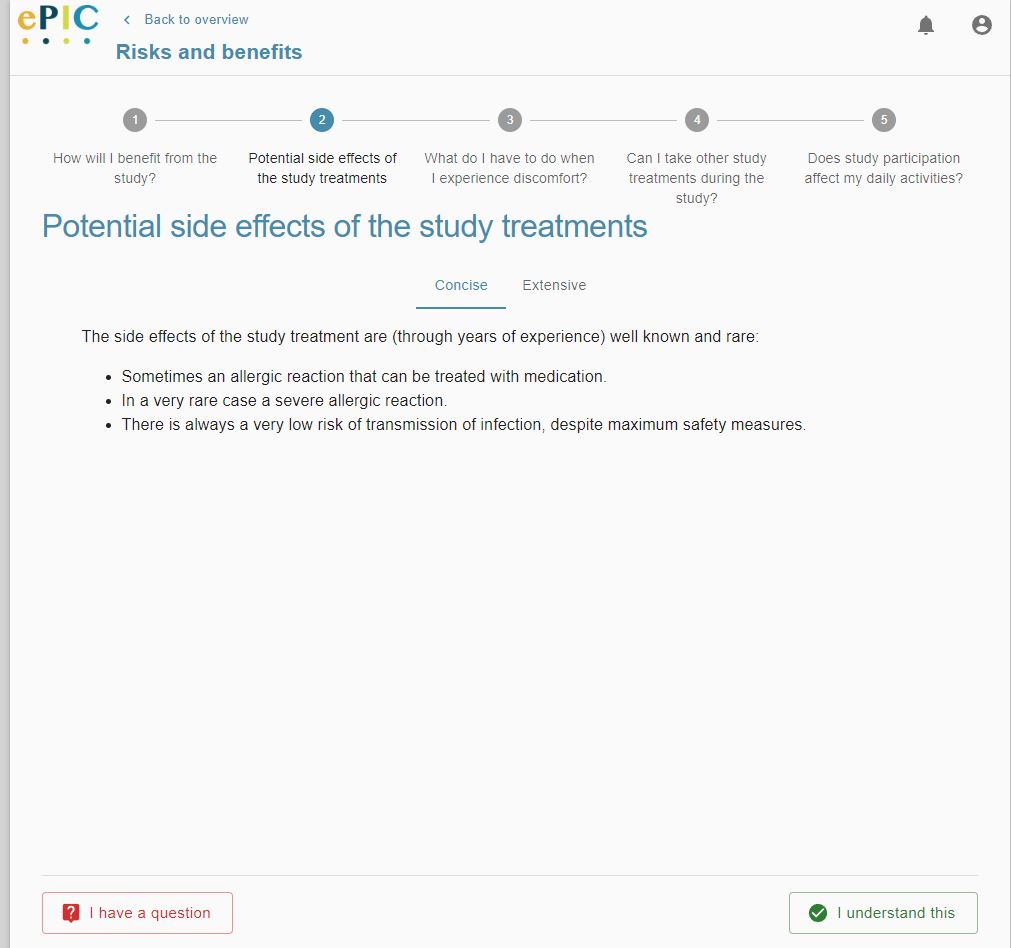


- The top of the interface includes the button ‘Back to overview’ (if this button is clicked, the participant navigates to interface 4).

**Interface 6: Your questions**

- This interface includes the headings ‘Make an appointment’ and ‘Overview’.
- Under the heading ‘Make an appointment’, the participant is informed that a video consultation with a member of the study team can be scheduled to discuss unclarities about the trial by making use of the button ‘Make an appointment for a video consultation’. Once scheduled, the video consultation can be started. In addition, the participant is informed that all unclarities must be solved to be able to sign the informed consent.
- Under the heading ‘Overview’, the sections and parts containing unclarities are listed with a red question mark icon. By clicking the button ‘Show’, the paragraph about which a participant has questions appears, accompanied by the participant’s notes. Hereafter, the participant has the possibility to click on the text buttons ‘Hide’, ‘Edit’, or ‘Mark as solved’. When the questions or unclarities are marked as solved, the red question mark icon changes into a green checkmark.


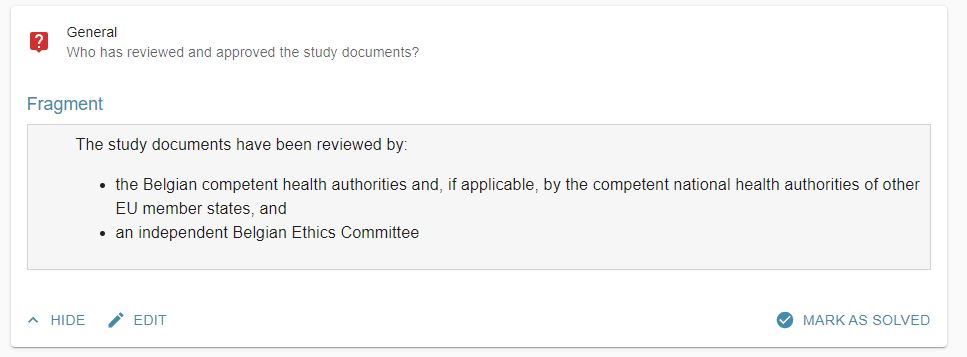


**Interface 7: Signing the consent**

- This interface contains a summary of study-related information and multiple signing options to provide consent.

**Interface 8: My profile**

- This interface contains the headings ‘Contact details’ and ‘Notifications’.
- Under the heading ‘Contact details’, the participant is able to enter or edit his/her email address and phone number.
- Under the heading ‘Notifications’, the participant can indicate, by making use of a slider, how he/she wishes to be informed about notifications of the platform or about new studies for which participants are being recruited.

**Interface 9: Study preferences**

- The participant can use a slider to indicate if he/she would like to be informed when interim results or a summary of the final results is made available. In addition, a hyperlink to the participants’ profile is included (i.e., interface 8).


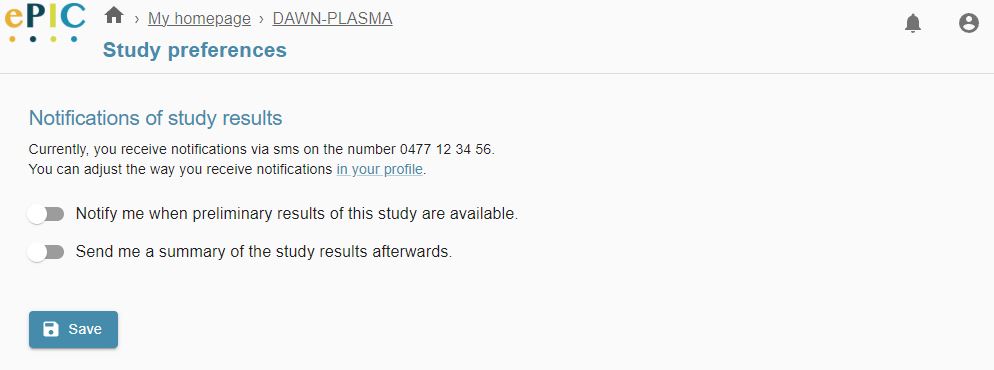


**Interface 10: Change or withdraw the consent**

- This interface contains the headings ‘Edit your choices’ and ‘Withdraw consent to participate’.
- Under the heading ‘Edit your choices’, the participant is able to change preferences (e.g., for data sharing) by selecting a radio button. The participant is informed that ‘*If you change these preferences, you will still participate in the study.*’.

Under the heading ‘Withdraw consent to participate’, the participant is able to withdraw his/her consent by clicking the red outlined button ‘Withdraw consent’. The participant is informed that ‘*If you withdraw your consent, you will no longer participate in this study. You cannot reverse this decision.*’.


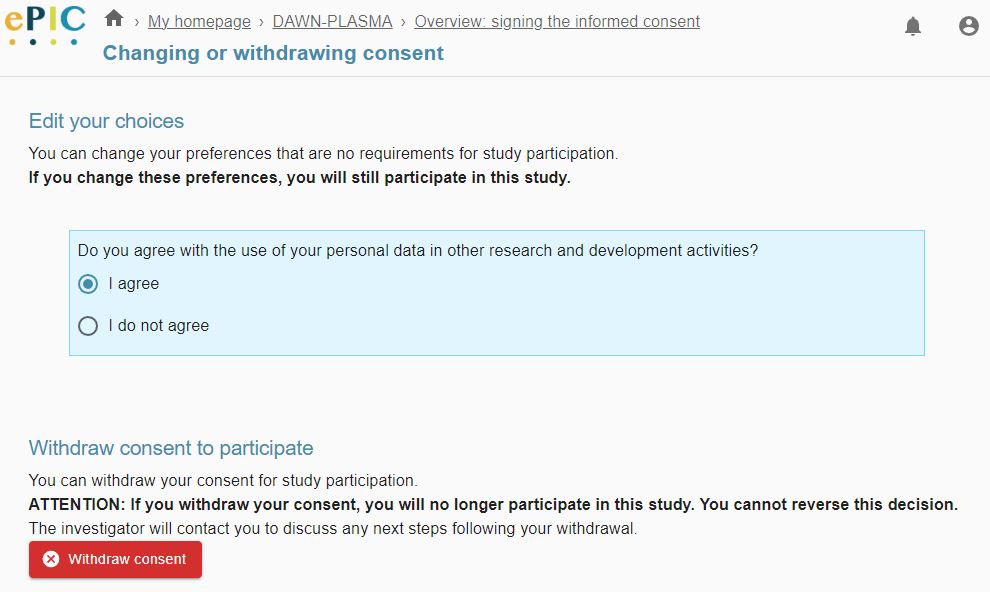

Supplement: Multimedia Appendix 2 [file jmir_v25i1e46306_app2.docx]
