# Supplementary material for: Testing and Practical Implementation of a User-Friendly Personalized and Long-Term Electronic Informed Consent Prototype in Clinical Research: Mixed Methods Study
Source: J Med Internet Res. 2023 Dec 19;25:e46306. doi: 10.2196/46306 (PMC10762617; doi:10.2196/46306)
Supplement: Multimedia Appendix 3 [file jmir_v25i1e46306_app3.docx]

**Multimedia Appendix 3. Survey including demographic questions and questions related to digital literacy**

Part 1: Experiences with information and communication technologies

**1. Do you have a computer or a tablet at home with internet access?**

Yes

No 🡪 *Go to Part 2: Personal characteristics*

**2. For which of the following activities do you use this computer or tablet?**

Internet banking

Seeking information

Sending / receiving emails

Consulting social media

Listening to music

Other

**3. How often on average did you use your computer or tablet in the last 3 months?**

Every day or almost every day, and even multiple times a day

Every day or almost every day, but not multiple times a day

At least once a week, but not (almost) every day

At least once a month, but not every week

Less than once a month

**4. For which of the following activities did you use your computer or tablet in the last 12 months? *(tick all that apply)***

Transferring files between computers, tablets, or other devices

Installing software or applications (apps)

Changing the settings of any software programs

None of the above

**5. Which of the following software activities did you carry out in the last 12 months? *(tick all that apply)***

Copying or moving files or folders

Using word processing software

Creating presentations or documents integrating text, pictures, tables, or charts

Using software to edit photos, video, or audio files

Writing code in a programming language

None of the above

Part 2: Personal characteristics

**6. Wat is your first and last name?**

**7. Wat is your age?**

**8. Wat is your sex?**

Male

Female

Other

I prefer not to answer

**9. What is your highest level of education?**

I did not complete secondary school

Secondary school or equivalent

Bachelor’s degree or equivalent

Master’s degree or equivalent

PhD

Other

**10. What is your employment situation?**

Student

Employed (full time, part time…)

Unemployed and not looking for work

Unemployed and looking for work

Retired

Other
