# Supplementary material for: Testing and Practical Implementation of a User-Friendly Personalized and Long-Term Electronic Informed Consent Prototype in Clinical Research: Mixed Methods Study
Source: J Med Internet Res. 2023 Dec 19;25:e46306. doi: 10.2196/46306 (PMC10762617; doi:10.2196/46306)
Supplement: Multimedia Appendix 4 [file jmir_v25i1e46306_app4.docx]

**Multimedia Appendix 4. Instructions for participants taking part in a usability test**

**Context for participants**

- **The purpose of this research study:** to inform the design of a user-friendly electronic informed consent interface, tailored to your needs.
- **We ask you to imagine that:**
  - You had taken part in a clinical study on **skin blood perfusion in humans in the past.**
  - A few days ago, you were informed that you are infected with the **Coronavirus** and that there is currently a clinical trial “**DAWN-PLASMA**” investigating treatments for this virus.
  - You have already had an interview with the investigator of this “DAWN-PLASMA” study. Based on this interview, you are interested in participating in this study. In order to take part, the investigator asked you to read the **information** regarding this clinical study and to provide your **informed consent** for participation.

**Instructions for participants**

- While reading the information regarding the “DAWN-PLASMA” study and providing your informed consent, you will complete various **computer tasks**.
- We ask you **to think aloud** while performing these tasks.
- After tasks 5 and 8, you will complete a **questionnaire**. In addition, a **short interview** will be conducted after each task.
- You, as a participant, will not be evaluated. It is therefore no problem if you take wrong actions. By performing computer tasks, you help us to **evaluate our user interface**.
