# Supplementary material for: Testing and Practical Implementation of a User-Friendly Personalized and Long-Term Electronic Informed Consent Prototype in Clinical Research: Mixed Methods Study
Source: J Med Internet Res. 2023 Dec 19;25:e46306. doi: 10.2196/46306 (PMC10762617; doi:10.2196/46306)
Supplement: Multimedia Appendix 5 [file jmir_v25i1e46306_app5.docx]

**Multimedia Appendix 5. Tasks for participants taking part in a usability test**

**Task 1:**

Consult the **symptoms** of an allergic reaction that may occur from the study treatment. You can find these symptoms in the section ‘Risks and benefits’. Say these symptoms out loud.

**Task 2:**

Consult the definition of the term **‘randomization’**. You can find this definition in the section ‘General’. Say this definition out loud.

**Task 3:**

Consult the organizations/persons who review your **coded data** sent to other countries within and outside the European Union. You can find this information in the section ‘Privacy and data protection’. Say one of these organizations/persons out loud.

**Task 4:**

a) Indicate that you do not understand this information, regarding **which organizations/persons review your coded data.**

b) Indicate that you have a question about **your preferences regarding the use of your data in other research and development activities.** *(for the first iteration only)*

c) Consult all information in the section ‘Privacy and data protection’. Indicate, where necessary, that you agree to the use of your data in other research and development activities. *(for the second and third iterations only)*

d) You have another question that you would like to discuss with the researcher. Please add the following question: “Will I always be in contact with the principal investigator?”. *(for the second iteration only)*

e) You have another question that you would like to discuss with the researcher. **This question does not belong to any of the sections.** Please add the following question: “Will I always be in contact with the principal investigator?”. *(for the third iteration only)*

f) Make an appointment for a video consultation with the research team to discuss unclarities. Say the message that you receive after making this appointment oud loud.

**Task 5:**

Imagine that we are several days later and that your video consultation will take place today.

a) Start this video consultation.

b) Imagine that your questions were answered. By the explanation of the research team you consent to the use of your data in other research and development activities. Please provide your informed consent for participation in this clinical study. Say the message that you receive after providing your consent out loud. Please log out. *(for the first iteration only)*

c) Imagine that your questions were answered. Please provide your informed consent for participation in this clinical study. Say the message that you receive after providing your consent out loud. Please log out. *(for the second and third iterations only)*

**Task 6:**

Imagine that we are several months later. You **no longer consent** to the use of your data in other research and development activities. Please make the necessary changes. Say the message that you receive after changing your preferences out loud.

**Task 7:**

a) Indicate that you would like to be contacted to receive a summary of the results of the COVID-19 study.

b) In the past, you indicated that you would like to receive this summary via text message. Please change this to **email**. Say the email address this will be sent to out loud.

**Task 8:**

a) You have been notified that a new informed consent version is available. Please look into this version. Say the number of sections that contain changes out loud.

b) Please identify what has changed about **the use of your data**. Provide as much information as possible. Say this information out loud.
