# Supplementary material for: Testing and Practical Implementation of a User-Friendly Personalized and Long-Term Electronic Informed Consent Prototype in Clinical Research: Mixed Methods Study
Source: J Med Internet Res. 2023 Dec 19;25:e46306. doi: 10.2196/46306 (PMC10762617; doi:10.2196/46306)
Supplement: Multimedia Appendix 6 [file jmir_v25i1e46306_app6.docx]

**Multimedia Appendix 6. Interview guide**

**I. Introductory question**

1. What is the practice of the ethics committee of which you are a member to assess a paper-based informed consent form? *(for ethics committee members only)*

2. What is your current practice to inform research participants and to obtain their consent? *(for health care professionals only)*

3. What are the sponsor’s responsibilities with regard to the informed consent process? *(for pharmaceutical industry representatives only)*

**II. Questions regarding electronic informed consent**

- Providing the definition issued by the European Medicines Agency: *“An electronic informed consent refers to the use of any digital media (e.g. text, graphics, audio, video, podcasts or websites) to firstly convey information related to the clinical trial to the trial participant and secondly document informed consent via an electronic device (e.g. mobile phones, tablets or computers).”*
- Introducing the electronic informed consent prototype: showing all functionalities related to personalization and the long-term interaction

The following questions (4-12) apply to ethics committee members only

4. What is your opinion on this prototype and on the integrated functionalities?

5. Which functionalities should be integrated in an electronic informed consent system to be an added value, compared to paper-based informed consent forms, for the ethics committee of which you are a member?

6. How would electronic informed consent influence the evaluation process for the ethics committee of which you are a member?

7. In what way would you like to assess the information presented in the electronic informed consent?

8. What is your opinion on keeping track of the ethics committee’s comments and decisions in an electronic informed consent system?

9. When a new informed consent version should be assessed based upon study amendments, how could an electronic informed consent system facilitate the assessment of this new version?

10. Which barriers or challenges may be experienced in the evaluation process of electronic informed consent?

11. Who should have access to an electronic informed consent system (i.e., the system and not specifically the signed electronic informed consent)?

12. What other systems should an electronic informed consent system ideally interact with?

The following questions (13-18) apply to health care professionals only

13. What is your opinion on this prototype and on the integrated functionalities? How would the use of this prototype influence your current practice?

14. In case you inform a digital literate participant and obtain this participant’s consent, how would the use of electronic informed consent influence your workload?

15. Which functionalities should be integrated in an electronic informed consent system to be an added value, compared to paper-based informed consent forms, for you as a health care professional involved in clinical research?

16. Which barriers or challenges may you experience when using electronic informed consent?

17. Who should have access to an electronic informed consent system (i.e., the system and not specifically the signed electronic informed consent)?

18. What other systems should an electronic informed consent system ideally interact with?

The following questions (19-23) apply to pharmaceutical industry representatives only

19. What is your opinion on this prototype and on the integrated functionalities? How would the use of this prototype influence your current practice?

20. Which functionalities should be integrated in an electronic informed consent system to be an added value, compared to paper-based informed consent forms, for a sponsor?

21. Which barriers or challenges may a sponsor experience when using electronic informed consent?

22. Who should have access to an electronic informed consent system (i.e., the system and not specifically the signed electronic informed consent)?

23. What other systems should an electronic informed consent system ideally interact with?
